# The clinical effect of Kampo medicine in multimodal treatment for Gastrointestinal Cancer in Japan

**DOI:** 10.7150/jca.46748

**Published:** 2020-07-11

**Authors:** Toru Aoyama, Hiroshi Tamagawa

**Affiliations:** Department of Surgery, Yokohama City University.

**Keywords:** Kampo medicine, gastrointestinal cancer, surgery, chemotherapy

## Abstract

Kampo medicine or Japanese/Chinese traditional herbal medicine has long been used for the treatment of various diseases, mainly in Asian countries. In recent years, Asian investigators have attempted to clarify the mechanism and clinical efficacy of Kampo medicine. This review summarizes the background, current status, and future perspectives of Kampo medicine in the multimodal treatment of gastrointestinal cancer. Regarding the clinical effect of Kampo medicine on postoperative dysfunction after gastrointestinal surgery, several investigators have reported that Daikenchuto (TJ-100) had clinical efficacy after abdominal digestive surgery. The administration of TJ-100 during the immediate postoperative period after esophageal cancer surgery, gastric cancer surgery, and liver cancer surgery appeared to promote early recovery of the postoperative bowel function. Regarding Kampo medicine for chemotherapy-induced adverse effects in gastrointestinal cancer, promising results have been obtained for Hangeshashinto (TJ-14) and Goshajinkigan (TJ-107). The addition of TJ-14 might be associated with an improvement in the duration of chemotherapy-induced oral mucositis, and the addition of TJ-107 might be associated with an improvement in oxaliplatin-induced peripheral neurotoxicity. However, while several clinical trials have shown the positive results of Kampo medication for gastrointestinal cancer treatment, the clinical effects of such medicines have been limited. Further trials to investigate the clinical benefits of Kampo medicine are needed.

## Introduction

An estimated 14.1 million new cancer cases and 8.2 million cancer deaths occurred in 2012 worldwide 1. Surgical resection is one of the major methods of treating gastrointestinal cancer 2-3. Chemotherapy is also an extremely important modality for managing advanced gastrointestinal cancer as well as for the curative resection of cancers in an adjuvant setting 6-9. Numerous regimens have been used for chemotherapy in cases of operable or inoperable gastrointestinal cancer. However, while several studies have shown that these treatments for gastrointestinal cancer prolongs the survival, such efforts often cause severe postoperative complications and toxicity, seriously compromising the patients' quality of life and precluding continuation of the treatment 6-9.

Kampo medicine or Japanese/Chinese traditional herbal medicine has long been used for the treatment of various diseases, mainly in Asian countries. The clinical effect of those Kampo medicines has been taken for granted, and their approval in western countries has been precluded due to two major reasons. First, Kampo medicines are mixtures of several ingredients to achieve a synergistic effect. Since the analysis of the efficacy of each ingredient is imperative in modern science, attempts to elucidate the mechanisms underlying Kampo medicines have not been entirely successful. Second, there have been few reliable, well-conducted clinical trials clarifying the efficacy of these approaches.

In recent years, Asian investigators have attempted to clarify the underlying mechanisms and clinical efficacy of Kampo medicine. This review summarizes the background, current status, and future perspectives of Kampo medicine in the multimodal treatment of gastrointestinal cancer.

## Clinical effects of Kampo medicine for postoperative dysfunction after gastrointestinal surgery

Surgical resection is a major method of treating gastrointestinal cancer. However, after surgery, patients experience general dysfunction, such as bowel and physical dysfunction, due to the long surgical duration and large abdominal incisions. Among Kampo medicines, several investigators have reported that Daikenchuto (TJ-100) showed clinical efficacy after abdominal digestive surgery 10-15. TJ-100 is a traditional Japanese medicine containing processed ginger, ginseng, and Zanthoxylum fruit. Since 2000, a number of studies have obtained evidence concerning its perioperative use in gastrointestinal surgery (**Table 1**).

In esophageal cancer surgery, Nishino et al. conducted a randomized trial in order to evaluate the efficacy of TJ-100 of postoperative dysfunction in patients with esophagectomy for esophageal cancer 16. The primary endpoints were maintenance of the nutrition condition and the recovery of the gastrointestinal function. Forty patients (TJ-100, n=20; control, n=20) were analyzed in this study. They showed that the percentage body weight loss at postoperative day 21 was significantly lower in the TJ-100 group than in the control group (TJ-100; 3.6% vs. control; 7.0%, p = 0.014). However, the serum albumin, level flatus, defecation, oral intake, and length of the hospital stay were not significantly different between the groups. They concluded that TJ-100 treatment after esophageal cancer resection had the effects of minimizing body weight loss and potentially suppressing the excess inflammatory reaction related to surgery. In gastric cancer, Yoshikawa et al. evaluated the efficacy of TJ-100 in treating gastrointestinal disorders in patients who received total gastrectomy for gastric cancer in a multicenter randomized controlled trial 17. Patients received either TJ-100 (15 g/day) or matching placebo from postoperative days 1 to 12. The primary endpoints were the time to first bowel movement (BM), frequency of BM, and time to first flatus. One hundred and ninety-five patient (TJ-100, n=96; placebo, n=99) were analyzed. The median time to first BM was marginally but significantly shorter in the TJ-100 group than in the placebo group (94.7 h vs 113.9 h, p=0.051). In addition, the incidence of more than 2 symptoms of gastrointestinal dysfunction was significantly lower in the TJ-100 group than in the placebo group on postoperative day 12 (p=0.026). That study concluded that the administration of TJ-100 during the immediate postoperative periods after total gastrectomy promoted the early recovery of the postoperative bowel function. In liver cancer, Shimada et al. conducted a multi-center phase III trial in order to evaluate the effect of TJ-100 on the anti-inflammatory response and liver function in patients with hepatic resection for liver cancer 18. The primary endpoints were the time to the first postoperative BM (FBM-T) and the serum C-reactive protein (CRP) and ammonia levels. Two hundred and nine patients (TJ-100, n=108; placebo, n=101) were analyzed in that study. The authors demonstrated that TJ-100 significantly accelerated the FBM-T compared to the placebo, as the median FBM-T was 88.2 h in the TJ-100 group and 93.1 h in the placebo group (p=0.0467). In contrast, the serum CRP and ammonia levels were similar between the TJ-100 group and placebo group. They concluded that TJ-100 is an effective treatment option for improving gastrointestinal dysmotility after hepatic resection.

However, despite these moderate clinical effects of Kampo medicine after gastrointestinal surgery, negative results have also been observed in some trials. For example, Okada et al. evaluated whether or not the perioperative administration of TJ-100 reduced the incidence postoperative paralytic ileus among patients with periampullary tumor or tumor of the head of the pancreas who received PD in a multicenter, randomized, double-blinded, placebo-controlled, phase II trial 19. In that study, the primary endpoints were the incidence of postoperative paralytic ileus lasting >72 h after surgery and the time to the occurrence of postoperative paralytic ileus. Two hundred and twenty-two patients underwent randomization; 112 received TJ-100, and 112 received placebo. Paralytic ileus occurred in 33.7% in the TJ-100 group and in 36.9% in the placebo group (p=0.626). In addition, the time to first flatus was 2.25 days (range: 2.00-2.50) in the TJ-100 group and 2.50 days (range: 1.50-2.50) in the placebo group (p=0.343). The authors concluded that the use of TJ-100 did not markedly improve the recovery from paralytic ileus after PD. Katsuno et al. also evaluated the efficacy of TJ-100 for accelerating the recovery of the gastrointestinal function in patients undergoing open colectomy for colon cancer in a multicenter randomized controlled trial 20. Patients received either TJ-100 (15 g/day) or a matching placebo from post-operative day 2 and post-operative day 8. The primary endpoints were the time to first BM, frequency of BM, and stool form. Three hundred and thirty-six patients (TJ-100, n=174; placebo, n=162) were analyzed. The time to first BM did not differ significantly between the TJ-100 group and placebo group. They concluded that their study did not adequately demonstrate the clinical benefits of TJ-100. In addition, Mizutani et al. conducted a randomized clinical trial to evaluate whether or not Inchinkoto (TJ-135) has postoperative hepatoprotective effects in patients who had undergone major hepatectomy 21. In the TJ-135 group, the patients received TJ-135 (15 g/day) for at least 7 days before surgery. The primary endpoint was the incidence of post-hepatectomy liver damage. Sixty-one patients (TJ-135, n=30; control, n=30) were analyzed. They found that the liver function test findings and incidence of post-hepatectomy liver failure did not differ significantly between the TJ-135 group and control group, suggesting that the preoperative administration of TJ-135 did not have a significant impact on the overall outcome of major hepatectomy**.**

Taken together, despite some positive results, especially concerning TJ-100 (Daikenchuto) after gastrointestinal surgery, these previous studies suggest that the clinical benefits of Kampo medicine after gastrointestinal surgery are limited.

## Clinical effects of Kampo medicine on chemotherapy-induced adverse effects for gastrointestinal cancer

Several trials have shown that chemotherapy is one of the most important treatment modalities for both operable and inoperable gastrointestinal cancers. However, while several studies have shown that chemotherapy prolongs the survival, it often causes severe toxicity, seriously compromising the patients' quality of life and precluding continuation of the treatment.

Recently, several randomized trials have shown that Kampo medicine prevents or ameliorates chemotherapy-induced adverse effects (**Table 2**). Regarding its effects on chemotherapy-induced oral mucositis (COM), Matsuda et al. conducted a double-blind, placebo-controlled, randomized comparative trial to investigate whether or not Hangeshashinto (TJ-14) prevents and controls COM in patients with colorectal cancer who develop moderate-to-severe COM during chemotherapy 22. Hangeshashinto is a traditional Japanese medicine containing a mixture of seven herbs (including pinellia tuber, scutellaria root, glycyrrhiza, jujube, ginseng, processed ginger, and coptis rhizome). Ninety eligible patients (TJ-14: 43, placebo: 47) were analyzed. Although the incidence of grade ≥2 oral mucositis was lower for patients treated with TJ-14 than in those treated with placebo, there was no significant difference (48.8% vs. 57.4%; p=0.41). In contrast, the incidence of grade ≥3 COM was 9.5% in the TJ-14 group and 17% in the placebo group. In addition, the median duration of grade ≥2 COM was 5.5 days vs. 10.5 days in the TJ-14 and placebo groups, respectively (p=0.018). No marked difference in other treatment toxicities was observed between the two groups. These study results did not meet the primary endpoint. However, TJ-14 demonstrated a significant effect in the treatment of grade ≥2 mucositis in patients with colorectal cancer compared to the placebo.

Similar results were observed in the gastric cancer region. We conducted a randomized comparative trial to investigate whether or not TJ-14 could prevent and control COM in gastric cancer 23. We randomly assigned 91 eligible patients with gastric cancer who developed moderate to severe oral mucositis (NCI-CTC grade ≥1) during any cycle of any chemotherapy to receive either TJ-14 or placebo (TJ-14: 45, placebo: 46) per the protocol set analysis after key opening. Patients then received placebo or TJ-14 at the start of the next course of chemotherapy. The treatment safety and presence of oral mucositis and its severity (using NCI-CTC grading) were assessed three times per week. The incidence of grade ≥2 oral mucositis was 40.0% in the TJ-14 group and 41.3% in the placebo group (p=0.588). The median duration of grade ≥2 mucositis was 14 days vs. 16 days in the TJ-14 and placebo groups, respectively (p=0.894). However, the median duration of grade ≥2 mucositis was 9 days vs. 17 days in the TJ-14 and placebo groups, respectively (p=0.290). Although this trial also did not reach its primary objective, the addition of TJ-14 to chemotherapy might be associated with an improvement in the duration of oral mucositis in patients with NCI-CTC Grade 1. A subgroup analysis also showed an increased benefit in patients with NCI-CTC Grade 1 oral mucositis.

Regarding oxaliplatin-induced peripheral neurotoxicity (OPN), Kono et al. conducted a double-blind, placebo-controlled, randomized comparative trial to investigate whether or not Goshajinkigan (TJ-107) prevents and controls OPN in patients with advanced or recurrent colorectal cancer (CRC) treated with the standard FOLFOX regimens 24. Goshajinkigan is a traditional Japanese medicine containing 10 herbal crude drugs (rehmanniae radix, achyranthis radix, corni fructus, moutan cortex, alismatis rhizome, dioscoreae rhizome, plantaginis semen, poria, processed aconiti tuber, and cinnamomi cortex). Eighty-nine eligible patients (TJ-107: 44, placebo: 45) were analyzed. The incidence of grade ≥2 OPN until the 8th cycle was 39% and 51% in the TJ-107 and placebo groups, respectively (relative risk, 0.76; 95% CI, 0.47-1.21). In addition, the incidence of grade 3 OPN was 7% vs. 13% in the TJ-107 and placebo groups, respectively (relative risk, 0.51, 0.14-1.92). They concluded that TJ-107 appears to have an acceptable safety margin and a promising efficacy in delaying the onset of grade ≥2 OPN without impairing the FOLFOX efficacy.

However, despite these studies demonstrating clinical efficacy in the advanced CRC setting, there have been some negative results in the adjuvant CRC setting. Oki et al. conducted a double blind, placebo-controlled, randomized comparative trial to investigate whether or not TJ-107 prevents and controls OPN in patients with colon cancer undergoing adjuvant therapy with infusion mFOLFOX6 regimen. One hundred and eighty-two patients (TJ-107: 89, placebo: 93) were analyzed 25. The incidence of grade ≥2 neurotoxicity was 50.6% in the TJ-107 group and 31.2% in the placebo group. They concluded that TJ-107 did not prevent OPN.

Given these previous findings, the clinical effects of Kampo medicine on chemotherapy-induced adverse effects appear to be controversial.

## Ongoing clinical trials of Kampo medicine for cancer treatment

Since 2015, several ongoing studies have examined various approaches with Kampo medicine for gastrointestinal cancer treatment. One study (University hospital Medical Information Network (UMIN) 000032697) is evaluating the safety and efficacy of the perioperative administration of TJ-100 in patients with postoperative functional aberration of the gastrointestinal tract due to surgery for colon cancer. This study is exploring whether or not the perioperative administration of TJ-100 affects the postoperative recovery of the gastrointestinal function in patients undergoing surgery for colon cancer. The primary endpoint is the time until the first flatus following colon surgery. Another trial (UMIN 000023318) is evaluating the safety and efficacy of TJ-100 for gastrointestinal symptoms, such as abdominal pain and distention, following laparoscopic colectomy in colon cancer patients. This study is exploring the effect of TJ-100 for the ethical use in treating accompanying abdominal symptoms and improving the quality of life after laparoscopic colectomy in colon cancer patients. The primary endpoints are the numerical rating scale of abdominal pain and abdominal distention and the gastrointestinal quality of life index. A third trial (UMIN000027561) is evaluating the efficacy of Yokukansan (TJ-54) for treating perioperative psychiatric symptoms in cancer patients undergoing highly invasive surgery. This trial is a randomized, double-blind, placebo-controlled trial to assess the efficacy and safety of TJ-54 on perioperative psychiatric symptoms in cancer patients. The primary endpoints are the change in the preoperative anxiety and incidence rate of postoperative delirium. A fourth trial (UMIN000025606) is evaluating the efficacy of Ninjin-Youei-To (TJ-108) for treating fatigue in patients with unresectable pancreatic cancer who receive nab-paclitaxel plus gemcitabine therapy. This study is exploring the efficacy of TJ-108 for treating fatigue in patients with unresectable pancreatic cancer who receive nab-paclitaxel plus gemcitabine therapy using several scores. The primary endpoint is the evaluation of fatigue and malaise using the Functional Assessment of Chronic Illness Therapy-Fatigue score until eight weeks after starting chemotherapy.

## Conclusions

Despite some clinical trials showing promising results concerning the efficacy of Kampo medicine for gastrointestinal cancer treatment, the clinical effects of Kampo medicine appear to be limited. Further trials investigating the clinical benefits of Kampo medicine are therefore needed.

## Figures and Tables

**Table 1:** Clinical effects of Kampo medicine for postoperative dysfunction after gastrointestinal surgery

Author (year)	Type of Cancer	Study medicine	Primary endpoint	Sample size	Results
Nishino (2016)	Esophageal cancer	Daikenchuto (TJ-100)	Maintenance of the nutrition condition, Recovery of the GI function	TJ-100,n=20 Control, n=20	Body weight loss at POD 21 was 3.6% in TJ-100 and 7.0% in control
Yoshikawa (2015)	Gastric cancer	Daikenchuto (TJ-100)	First bowel movement (BM), Frequency of BM, and Time to first flatus	TJ-100,n=96; Placebo, n=99	Median time to first BM was 94.7 h inTJ-100 and 113.9 h in placebo
Shimada (2015)	Liver Cancer	Daikenchuto (TJ-100)	First postoperative BM (FBM-T),the serum CRP and ammonia levels	TJ-100,n=108; Placebo, n=101	FBM-T was 88.2 h in TJ-100 and 93.1 h in the placebo group
Okada (2016)	Pancreatic head tumor	Daikenchuto (TJ-100)	Incidence of paralytic ileus	TJ-100,n=112 Placebo, n=112	Paralytic ileus occurred in 33.7% in TJ-100 and in 36.9% in placebo
Katsuno (2015)	Colon cancer	Daikenchuto (TJ-100)	Time to first BM, frequency of BM, and stool form	TJ-100, n=174; Placebo, n=162	Time to first BM did not differ between the TJ-100 and placebo
Mizutani (2015)	Major hepatectomy	Inchinkoto (TJ-135)	Incidence of post-hepatectomy liver damage	TJ-135,n=30 Control, n=30	Incidence of post-hepatectomy liver failure did not differ between TJ-135 and control

**Table 2:** Clinical effects of Kampo medicine on chemotherapy-induced adverse effects for gastrointestinal cancer

Author (year)	Type of Cancer	Study medicine	Primary endpoint	Sample size	Results
Matsuda (2015)	Colorectal cancer	Hangeshashinto (TJ-14)	Incidence of chemotherapy-induced oral mucositis (COM)	TJ-14,n=43 Placebo, n=47	COM was 48.8% in TJ-14 and 57.4% in placebo
Aoyama (2014)	Gastric cancer	Hangeshashinto (TJ-14)	Incidence of chemotherapy-induced oral mucositis (COM)	TJ-14,n=45 Placebo, n=46	COM was 40.0% in TJ-14 and 41.3% in placebo
Kono (2013)	Colorectal cancer	Goshajinkigan (TJ-107)	Incidence of oxaliplatin-induced peripheral neurotoxicity (OPN)	TJ-107, n=44 Placebo, n=45	OPN was 39% in TJ-14 and 51% in placebo
Oki (2015)	Colorectal cancer	Goshajinkigan (TJ-107)	Incidence of oxaliplatin-induced peripheral neurotoxicity (OPN)	TJ-107, n=89 Placebo, n=93	OPN was 50.6% in TJ-14 and 31.2% in placebo
